# Serum IL-23 significantly decreased in obese patients with psoriatic arthritis six months after a structured weight loss intervention

**DOI:** 10.1186/s13075-023-03105-8

**Published:** 2023-07-27

**Authors:** A. J. Landgren, C. A. Jonsson, A. Bilberg, B. Eliasson, L. Torres, M. Dehlin, L. T. H. Jacobsson, I. Gjertsson, I. Larsson, E. Klingberg

**Affiliations:** 1 Department of Rheumatology and Inflammation Research, Institute of Medicine, Sahlgrenska Academy, University of Gothenburg, Gothenburg, Sweden; 2 Region Västra Götaland, Research and Development Primary Health Care, Gothenburg, Södra Bohuslän, Sweden; 3 Institute of Neuroscience and Physiology, Section of Health and Rehabilitation, Physiotherapy, Sahlgrenska Academy, University of Gothenburg, Gothenburg, Sweden; 4 Department of Rheumatology, Sahlgrenska University Hospital, Gothenburg, Sweden; 5 Department of Medicine, Sahlgrenska University Hospital, Gothenburg, Sweden; 6 Department of Gastroenterology and Hepatology, Sahlgrenska University Hospital, Institute of Medicine, Sahlgrenska Academy, University of Gothenburg, Gothenburg, Sweden

**Keywords:** Adipokines, Cytokines, Obesity, Psoriatic arthritis

## Abstract

**Introduction:**

Patients with psoriatic arthritis (PsA) are frequently obese. We have previously shown decreased disease activity in patients with PsA with a body mass index (BMI) ≥ 33 kg/m^2^ following weight loss treatment with Very Low Energy Diet (VLED), resulting in a median weight loss of 18.6% at six months (M6) after baseline (BL). In this study we assessed the effects of VLED on cytokines and adipokines at M6 in the same patients with PsA and controls (matched on sex, age and weight).

**Methods:**

VLED (640 kcal/day) during 12 or 16 weeks, depending on BL BMI < 40 or ≥ 40 kg/m^2^, was taken and followed by an energy-restricted diet. Cytokines and adipokines were measured with Magnetic Luminex Assays at BL and M6.

**Results:**

Serum interleukin (IL)-23, (median (interquartile range) 0.40 (0.17–0.54) ng/mL vs. 0.18 (0.10–0.30) ng/mL, *p* < 0.001) and leptin (26.28 (14.35–48.73) ng/mL vs. 9.25 (4.40–16.24) ng/mL, *p* < 0.001) was significantly decreased in patients with PsA. Serum total (tot)-adiponectin and high molecular weight (HMW) adiponectin increased significantly. Similar findings were found in controls. Also, in patients with PsA, ∆BMI was positively correlated with ∆IL-23 (r_S_ = 0.671, *p* < 0.001). In addition, significant positive correlations were found between ΔBMI and ΔDisease Activity Score (DAS28CRP), ΔCRP, Δtumor necrosis factor (TNF)-α, ΔIL-13, ∆IL-17 and Δleptin, and negative correlations between ΔBMI and Δtot-adiponectin.

**Conclusions:**

Weight loss was associated with decreased levels of leptin and cytokines, in particular IL-23. These findings may partly explain the anti-inflammatory effect of weight reduction in PsA.

**Trial registration:**

ClinicalTrials.gov identifier: NCT02917434, registered on September 21, 2016, retrospectively registered.

**Supplementary Information:**

The online version contains supplementary material available at 10.1186/s13075-023-03105-8.

## Background

Psoriatic arthritis (PsA) is a chronic inflammatory joint disease, characterized by psoriasis (PsO), arthritis, enthesitis and sometimes spondyloarthritis [1]. Patients with PsA have an increased cardiovascular risk due to the chronic inflammation and increased prevalence of obesity (Body Mass Index (BMI)) ≥ 30 kg/m^2^), hypertension and diabetes compared to individuals without PsA [2, 3]. Obesity is associated with augmented risk of PsA [4], more severe disease [5] and poorer treatment outcomes [6–8]. In obesity, the white adipose tissue undergoes immunological changes including macrophage infiltration resulting in a proinflammatory milieu and secretion of several cytokines and adipokines, such as tumor necrosis (TNF)-α, interleukin (IL)-17, IL-23, leptin, resistin and adiponectin, that may be important in PsA [9–11]. We have previously shown that in 41 patients with PsA and BMI ≥ 33 kg/m^2^, weight loss through Very Low Energy Diet (VLED) resulted in a median weight loss of 18.6% of baseline (BL) weight and significant improvements in Disease Activity in PSoriatic Arthritis (DAPSA and Disease Activity Score based on 28 joints using CRP (DAS28CRP) at six months (M6) [12].

To the best of our knowledge, no previous study has assessed the effects of weight loss on cytokines and adipokines in PsA. This study aimed to determine the effects of weight loss treatment with VLED on cytokines and adipokines at M6 in patients with PsA and obesity. A control group (matched on sex, age and weight) and without rheumatic disease or PsO that underwent the same treatment was included as comparators.

## Methods

### Study design and setting

This is an open prospective interventional weight loss study for which the study method has previously been described thoroughly [12, 13]. The study was conducted in Western Sweden, Gothenburg, at the Department of Rheumatology and the Regional Obesity Center at Sahlgrenska University hospital.

### Patients with PsA

Patients with PsA were recruited from hospitals in Western Sweden, including the Department of Rheumatology at Sahlgrenska University Hospital and rheumatology units at the hospitals of Borås and Alingsås, Sweden. Patients between 25–75 years of age, with BMI ≥ 33 kg/m^2^, and that fulfilled the Classification criteria for Psoriatic Arthritis (CASPAR) criteria [14] were eligible for inclusion. Exclusion criteria for patients and controls were: epilepsy, pregnancy, porphyria, type 1 diabetes, severe kidney, heart, or catabolic disease, binge eating disorder, current treatment with lithium, warfarin, or phenytoin, having mental imbalance affecting participation, a history of a myocardial infarction, stroke, major surgery or trauma during the last three months, or cancer treatment during the last five years. Conventional synthetic (cs) or biologic disease-modifying anti-rheumatic drugs (bDMARDs) were held unchanged from three months before BL until M6.

### Controls

Individuals with obesity that were already planned to undergo VLED treatment were recruited as controls from the Regional Obesity Center at Sahlgrenska University Hospital and matched for sex, age and weight to patients with PsA. Exclusion criteria were having a diagnosis of PsO, PsA or any inflammatory rheumatic disease in addition to the exclusion criteria for the patients with PsA.

### Ethics

All participants gave their written informed consent. The study was approved by the Regional Ethics Committee in Gothenburg (approval number 901–15). A patient representative was involved in the planning and design of the study.

### The dietary intervention

VLED (640 kcal/day) (Cambridge Weight Plan Limited, Corby, UK), was given to patients with PsA and controls, by study nurses for 12 or 16 weeks, depending on whether BL BMI < 40 or ≥ 40 kg/m^2^. After this period, food was gradually reintroduced during 12 weeks. Each participant was given personalised energy-restricted dietary advice by study dietitians. All participants were followed at the Regional Obesity Center during 12 months in accordance with the routines for structured weight loss treatment. All participants were seen by a physiotherapist at the Department of Rheumatology at BL and after six and twelve months and received the advice to engage in health improving physical activity ≥ 150 min per week, according to general Swedish recommendations.

### Physical and survey assessments

The patients with PsA were followed at the Clinical Rheumatologic Research Unit at Sahlgrenska University Hospital at BL, after three and after six months. The controls were followed with a similar protocol. Body height and weight was measured with a calibrated ruler and a digital scale and BMI was calculated. Waist circumference was measured in standing position with a tape measure midway between the lower rib and iliac crest. In patients with PsA, joints (66/68 swollen/tender joints count), entheses (Leeds enthesitis index) [15] and activity limitations (Health Assessment Questionnaire (HAQ)) [16] were assessed. The DAS28CRP and the DAPSA score were calculated [17, 18]. The extent of PsO was evaluated with body surface area (BSA) [19]. The patients’ disease activity was assessed with visual analogue scales (VAS) for global disease activity, pain and fatigue. Minimal disease activity (MDA) was defined as meeting minimum five of seven criteria: tender joint count 68 ≤ 1, swollen joint count 66 ≤ 1, psoriasis BSA ≤ 3%, patient pain VAS ≤ 15 mm, patient´s global disease activity VAS ≤ 20 mm, HAQ ≤ 0.5 and tender entheseal points ≤ 1 [20].

### Laboratory assessments

Blood samples were drawn from the participants in the morning after ≥ 8 h of fasting and stored at -80 °C. C-reactive protein (CRP) was analyzed by standard techniques at the Sahlgrenska University Hospital. Serum levels of cytokines and adipokines were assessed at BL and M6. The serum levels of the cytokines (TNF-α, IL-1β, IL-6, IL-8, IL-12/IL-23 p40, IL-13, IL-17, IL-23, interferon (IFN)-γ, and the adipokines (leptin (analyzed at twofold dilutions), resistin, total (tot)-adiponectin (200-fold dilution) and High Molecular Weight (HMW)-adiponectin (50-fold dilution)) were measured with Human Magnetic Luminex® Assays (R&D-systems) at the Department of Rheumatology and Inflammation research, Gothenburg University, according to the manufacturer's instructions. The analysis and quantification were performed using a Bio-Plex 200 system (Bio-Rad) with five-parameter logistic standard curves. Samples with analyte levels below the detection level were excluded from the analysis.

### Statistical analyses

Descriptive statistics are presented as numbers (%), median and interquartile range (IQR). The Mann–Whitney U test was used for comparisons of continuous variables between groups. The chi-square test was used for categorical variables. Wilcoxon Signed Rank Test was used to compare continuous related samples. Correlations were calculated using Spearman’s correlation (r_S_). Two-tailed tests were used and *p* ≤ 0.05 was considered statistically significant. Statistical analyses were made using SPSS Statistics version 29 (IBM, Chicago, USA). Figure 2 was made using R, version 4.1.2.

## Results

### Baseline

In total, 41 patients (median age 54 (IQR 49–62) years; 63% women) and 39 controls (median age 55 (IQR 46–60) years; 74% women) were included in this study regarding serum levels of cytokines and adipokines. The BL characteristics are shown in Table 1. There were no significant differences in height, weight or waist circumference comparing patients with PsA and controls, BMI was, however, lower in the patients with PsA, 35.2 (IQR 34.1–38.1) kg/m^2^ compared with the controls, 37.7 (36.7–41.5) kg/m^2^, *p* = 0.001. Serum IL-23 levels were lower in the patients with PsA (0.40 (0.17–0.54) ng/mL vs 0.54 (0.33–0.71) ng/mL, *p* = 0.027) than in controls, whereas serum IL-17 levels were higher in patients with PsA compared to controls, (2.63 (1.58–4.65) pg/mL vs. 0.86 (0.41–3.35) pg/mL, *p* = 0.022). The concentrations of IL-17 in serum, were however in general very low and should therefor be interpreted with caution. Number of individuals with levels below the detection limit were: PsA at BL, *n* = 21, PsA at M6, *n* = 18, controls at BL, *n* = 19, controls at M6, *n* = 20.

**Table 1:** Comparison of characteristics between patients with psoriatic arthritis and controls at baseline

	PsA, *n* = 41	Controls, *n* = 39	*p*-value
Women	26 (63.4)	29 (74.4)	0.424
Age, years	54 (49–62)	55 (46–60)	0.458
Body weight, kg	106.3 (95.8–113.6)	104.6 (96.5–120.0)	0.690
Height, m	1.68 (1.62–1.77)	1.66 (1.62–1.72)	0.190
BMI, kg/m^2^	35.2 (34.1–38.1)	37.7 (36.7–41.5)	**0.001**
Waist circumference, cm	116 (112–122)	117 (107–126)	0.817
Current smoking	1 (2.4)	2 (5.1)	0.527
SJC 66, score	0 (0–1)		
TJC 68, score	4 (1–14)		
VAS patients´ global disease activity, mm	34 (19–61)		
VAS pain, mm	30 (19–63)		
VAS fatigue, mm	56 (22–67)		
PsO duration, years	32 (19–40)		
PsA symptoms duration, years	17 (11–27)		
PsA peripheral disease	35 (85)		
Axial disease	2 (5)		
Combination peripheral/axial disease	4 (10)		
Dactylitis ever	21 (51)		
DAPSA, score	15.3 (6.6–29.1)		
DAS28CRP, score	2.9 (2.1–3.7)		
BSA, %	0.8 (0–2.3)		
Leeds enthesitis index	1 (0–4)		
NSAIDs	26 (63)		
csDMARD without bDMARD	19 (46)		
bDMARD	16 (39)		
TNF-inhibitor	15 (37)		
Methotrexate monotherapy	13 (31.7)		
Apremilast	1 (2.4)		
Prednisone	3 (7.3)		
Ustekinumab monotherapy	1 (2.4)		
IL-17- and IL-23-inhibitors	0		
CRP, mg/L	4.0 (2.0–8.5)	4.0 (2.0–6.0)	0.280
**Serum concentrations of cytokines and adipokines**			
TNF-α, pg/mL	12.92 (9.99–17.20)	11.66 (8.49–13.54)	0.077
IL-1β, pg/mL	12.86 (6.73–19.69)^c^	12.13 (5.30–14.81)^d^	0.399
IL-6, pg/mL	8.72 (6.23–11.18)	7.23 (5.54–9.47)	0.154
IL-8, pg/mL	18.69 (13.55–23.21)	19.28 (13.86–27.32)	0.397
IL-12/IL-23 p40, pg/mL	602.65 (384.93–900.21)^a^	544.11 (333.38–835.83)^c^	0.626
IL-13, pg/mL	906.55 (679.96–1131.63)^a^	885.07 (650.51–1103.93)^b^	0.501
IL-17, pg/mL	2.63 (1.58–4.65)^f^	0.86 (0.41–3.35)^e^	**0.022**
IL-23, ng/mL	0.40 (0.17–0.54)	0.54 (0.33–0.71)	**0.027**
IFN-γ, pg/mL	61.98 (43.73–76.34)	54.73 (43.83–69.95)	0.289
Resistin, ng/mL	12.83 (10.58–15.77)	11.82 (9.22–16.42)	0.427
Leptin, ng/mL	26.28 (14.35–48.73)	38.80 (20.47–59.87)	0.059
HMW adiponectin, µg/mL	3.39 (2.13–5.12)	4.26 (2.07–6.45)	0.384
Tot-adiponectin, µg/mL	4.03 (3.18–6.06)	4.16 (3.27–5.11)	0.832

Numbers are median (interquartile range) or n (%)

Number of patients with missing data values;

^a^ = 1 ^b^ = 2 ^c^ = 10 ^d^ = 11 ^e^ = 19 ^f^ = 21

*BMI* body mass index, *BSA* body surface area, *CRP* c-reactive protein, *Cs-DMARD* conventional synthetic disease modifying anti rheumatic drug, *DAPSA* disease activity in psoriatic arthritis, *DAS28* disease activity score, *HAQ* health assessment questionnaire, *HMW* high molecular weight, *IFN* interferon, *IL* interleukin, *NSAID* non-steroidal anti-inflammatory drug, *PsA* psoriatic arthritis, *PsO* psoriasis, *SJC* swollen joint count, *TJC* tender joint count, *TNF* tumor necrosis factor, *Tot* total, *VAS* visual analogue scale

In patients with PsA the median DAPSA score at BL was 15.3 (6.6–29.1) indicating moderate disease activity, and the majority (35/41, 85%) of patients with PsA had peripheral disease. Sixteen (39%) reported use of bDMARD and around half (19/41, 46%) reported use of csDMARD with no bDMARD. At BL, *n* = 12 (29.3%) of patients with PsA fulfilled criteria for MDA whereas *n* = 29 (70.7%) did not.

PsA patients with MDA at BL had lower levels of serum IL-23 (0.17 (0.08–0.35) ng/mL vs 0.50 (0.28–0.62) ng/mL, *p* = 0.002), leptin (16.9 (10.7–25.5) ng/mL vs 36.4 (15.7–52.6) ng/mL, *p* = 0.039) and BMI (34.5 (33.3–35.5) kg/m^2^, vs 36.0 (34.5–39.3) kg/m^2^, *p* = 0.036) compared to patients without MDA. There were no other significant BL differences regarding cytokines and adipokine levels. When instead stratifying the 41 patients with PsA by usage/non-usage of bDMARD, no significant differences in cytokines and adipokines were evident.

### Cytokines and adipokines at M6 compared to BL

**Fig. 1:**
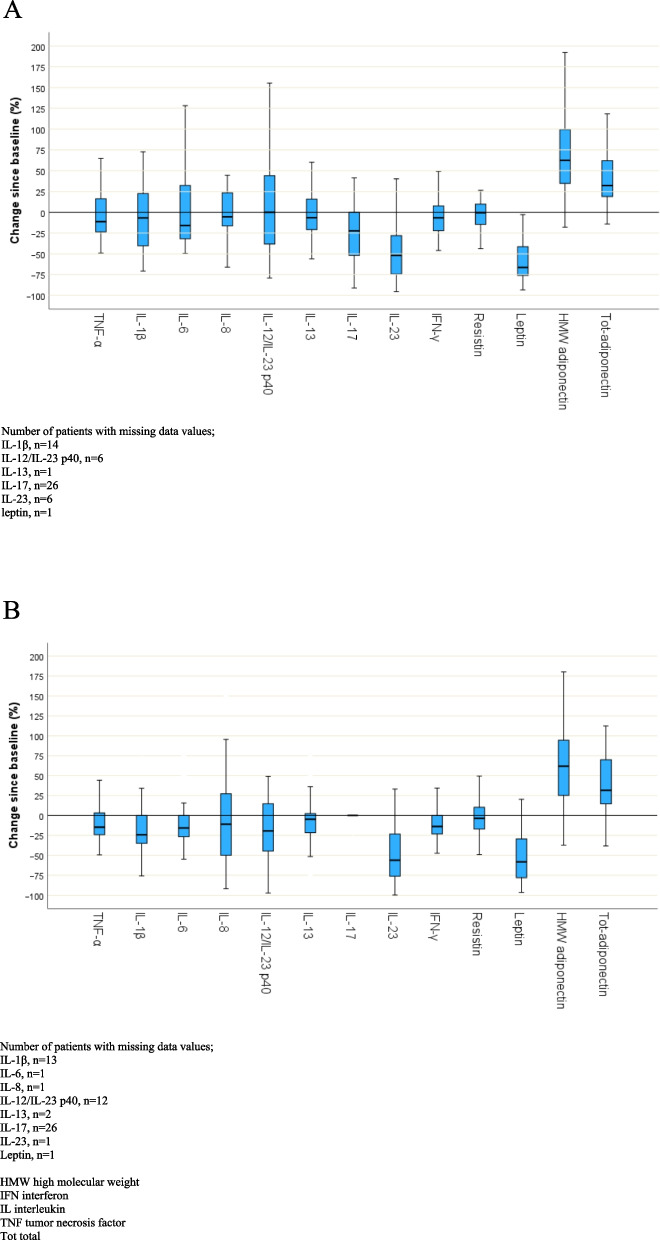
**A**, Baseline (BL) to month 6 (M6) change (%) in patients with psoriatic arthritis (PsA) (*n* = 41), **B**, Baseline (BL) to month 6 (M6) change (%) in controls (*n* = 39) Boxplots for cytokines and adipokines, showing the distribution of change (median and interquartile range) in %, comparing baseline (BL) and month 6 (M6)

**Fig. 2:**
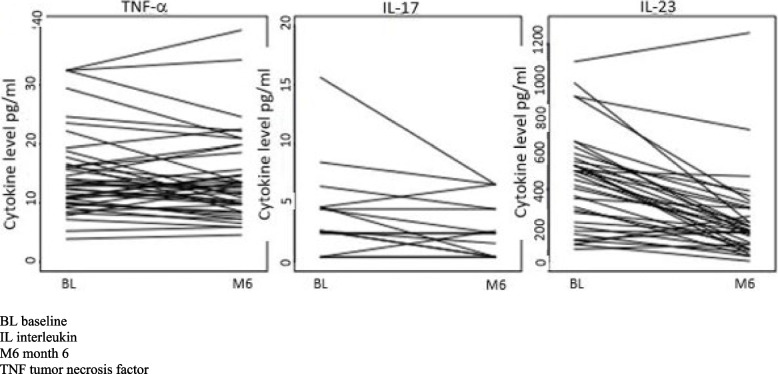
Serum levels of TNF-α, IL-17 and IL-23 in patients with psoriatic arthritis at baseline and at 6 months

Results are shown in Table 2 and Fig. 1A-B. A weight loss of 18.9 (15.0–26.5) kg in patients with PsA and 22.6 (14.8–28.5) kg in controls was noted at M6. There were no significant differences in BMI at M6 comparing patients with PsA and controls. In the patients with PsA, a significant decrease was noted in serum IL-23, 0.40 (0.17–0.54) ng/mL at BL to 0.18 (0.10–0.30) ng/mL at M6, *p* < 0.001. Serum leptin decreased from 26.28 (14.35–48.73) ng/mL to 9.25 (4.40–16.24) ng/mL, *p* < 0.001. Conversely, significantly increased serum levels of tot-adiponectin and HMW adiponectin were seen. In Fig. 2, serum levels of TNF-α, IL-17 and IL-23 for individual patients with PsA at BL and M6 are shown. In controls, serum levels of TNF-α, IL-1β, IL-6, IL-12/IL-23 p40, IL-23, IFN-γ and leptin, decreased significantly whereas serum tot-adiponectin and HMW adiponectin increased significantly at M6 (Table 2, Fig. 1A-B).

**Table 2:** Comparison of values at baseline (BL) and after 6 months (M6) in patients with psoriatic arthritis (PsA) and controls

Analytes	PsA (BL), *n* = 41	PsA (M6), *n* = 41	*p*-value PsA, BL vs M6	Controls (BL), *n* = 39	Controls (M6), *n* = 39	*p*-value controls, BL vs M6
TNF-α, pg/mL	12.92 (9.99–17.20)	12.49 (9.13–17.09)	0.234	11.66 (8.49–13.54)	9.60 (7.84–13.01)	**0.003**
IL-1β, pg/mL^a^	12.86 (6.73–19.69)	10.53 (5.02–19.69)	0.231	12.13 (5.30–14.81)	8.61 (5.30–14.28)	**0.048**
IL-6, pg/mL^b^	8.72 (6.23–11.18)	7.53 (5.34–10.03)	0.444	7.23 (5.54–9.47)	6.90 (4.92–8.33)	**0.005**
IL-8, pg/mL^c^	18.69 (13.55–23.21)	17.68 (13.53–21.65)	0.781	19.28 (13.86–27.32)	16.64 (11.85–19.41)	0.077
IL-12/IL-23 p40^d^, pg/mL	602.65 (384.93–900.21)	666.57 (333.38–974.17)	0.978	544.11 (333.38–835.83)	466.00 (209.26–729.91)	**0.032**
IL-13, pg/mL^e^	906.55 (679.96–1131.63)	885.07 (561.48–1171.12)	0.377	885.07 (650.51–1103.93)	777.97 (551.40–1023.60)	0.053
IL-17, pg/mL^f^	2.63 (1.58–4.65)	2.43 (0.41–4.48)	0.074	0.86 (0.41–3.35)	0.41 (0.41–2.63)	0.465
IL-23, ng/mL^g^	0.40 (0.17–0.54)	0.18 (0.10–0.30)	< **0.001**	0.54 (0.33–0.71)	0.23 (0.12–0.34)	< **0.001**
IFN-γ, pg/mL	61.98 (43.73–76.34)	52.09 (40.50–76.34)	0.134	54.73 (43.83–69.95)	48.80 (40.50–61.98)	**0.006**
Resistin, ng/mL	12.83 (10.58–15.77)	11.81 (10.05–14.91)	0.341	11.82 (9.22–16.42)	12.45 (9.19–15.04)	0.315
Leptin, ng/mL^h^	26.28 (14.35–48.73)	9.25 (4.40–16.24)	< **0.001**	38.80 (20.47–59.87)	14.04 (10.07–26.92)	< **0.001**
HMW adiponectin, µg/mL	3.39 (2.13–5.12)	5.95 (3.78–8.45)	< **0.001**	4.26 (2.07–6.45)	5.78 (4.50–7.93)	< **0.001**
Tot-adiponectin, µg/mL	4.03 (3.18–6.06)	5.90 (4.04–7.93)	< **0.001**	4.16 (3.27–5.11)	5.67 (4.27–6.70)	< **0.001**
VAS patients´ global disease activity, mm	34 (19–61)	12 (5–51)	**0.001**			
VAS pain, mm	30 (19–63)	20 (5–52)	**0.004**			
VAS fatigue, mm	56 (22–67)	25 (8–44)	**0.001**			
HAQ, score	0.63 (0.13–1.00)	0.25 (0.00–0.69)	< **0.001**			
DAS28CRP, score	2.9 (2.1–3.7)	2.5 (1.7–3.0)	< **0.001**			
DAPSA, score	15.3 (6.6–29.1)	8.2 (2.8–17.7)	< **0.001**			
BMI, kg/m^2^	35.2 (34.1–38.1)	29.8 (26.6–31.5)	< **0.001**	37.7 (36.7–41.5)	30.4 (27.9–33.2)	< **0.001**
CRP, mg/L	4.0 (2.0–8.5)	2.0 (1.0–6.5)	**0.041**	4.0 (2.0–6.0)	2.0 (1.0–4.0)	< **0.001**

Values are median (interquartile range) or n (%)

*p*-value for comparison between BL and M6 are based on cytokine levels above detection level, present in:

^a^31 patients with PsA and 28 controls, ^b^41 patients with PsA and 38 controls, ^c^41 patients with PsA and 38 controls, ^d^35 patients with PsA and 29 controls, ^e^40 patients with PsA and 37 controls, ^f^20 patients with PsA and 19 controls, ^g^35 patients with PsA and 38 controls, ^h^40 patients with PsA and 38 controls

*BL* baseline, *BMI* body mass index, *CRP* c-reactive protein, *DAPSA* disease activity in psoriatic arthritis, *DAS28CRP* disease activity score, *HAQ* health assessment questionnaire, *HMW* high molecular weight, *IFN* interferon, *IL* interleukin, *M6* month 6 after baseline, *TNF* tumor necrosis factor, *Tot* total, *VAS* visual analogue scale

### Change in cytokines and adipokines in relation to change in other variables in patients with PsA

**Fig. 3:**
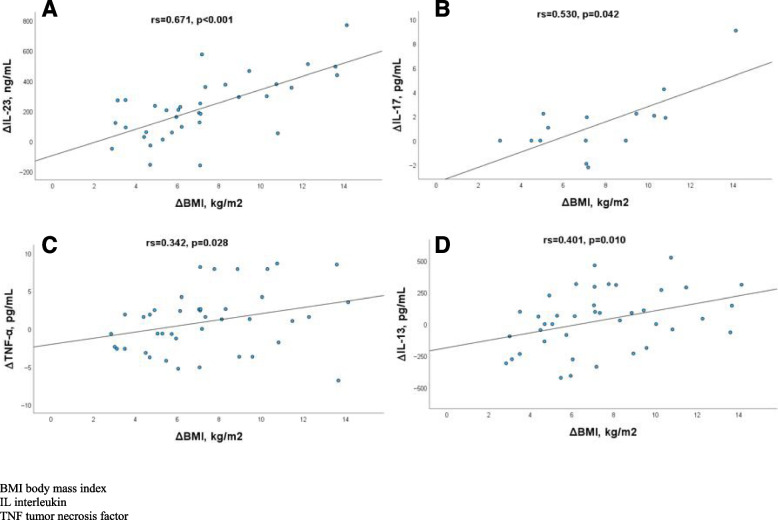
Scatterplots showing correlations between changes in BMI and in cytokine levels between baseline and 6 months in patients with psoriatic arthritis: **a** (∆IL-23 and ∆BMI), **b** (∆IL-17 and ∆BMI), **c** (∆TNF-α and ∆BMI), **d** (∆IL-13 and ∆BMI)

In patients with PsA, ∆BMI was positively correlated with ∆IL-23 (r_S_ = 0.671, *p* < 0.001) and ∆IL17 (r_S_ = 0.530, *p* = 0.042). Positive correlations were found between ∆BMI and ∆DAS28CRP, ∆CRP, ΔTNF-α, ΔIL-13, Δleptin whereas ∆BMI negatively correlated with Δtot-adiponectin (Table 3 and Fig. 3).

**Table 3:** Spearman correlations for cytokines, adipokines, disease activity measurements and BMI between baseline (BL) and 6 months (M6) of follow-up in patients with psoriatic arthritis (PsA), *n = 41*

	**ΔBMI**	***p*-value**	**ΔDAS28CRP**	***p*-value**	**ΔDAPSA**	***p*-value**	**ΔCRP**	***p*-value**
ΔBMI			0.450	**0.003**	0.178	0.264	0.336	**0.032**
∆TNF-α	0.342	**0.028**	0.303	0.054	0.272	0.085	0.144	0.371
∆IL-1β	0.263	0.184	0.234	0.241	0.220	0.271	0.056	0.781
∆IL-6	0.082	0.610	0.115	0.474	0.161	0.316	0.249	0.116
∆IL-8	-0.140	0.382	-0.094	0.559	-0.094	0.561	-0.134	0.404
∆IL-12/IL-23 p40	0.294	0.087	0.260	0.132	0.307	0.073	-0.044	0.802
∆IL-13	0.401	**0.010**	0.232	0.150	0.257	0.110	-0.174	0.284
∆IL-17	0.530	**0.042**	0.448	0.094	0.209	0.454	0.204	0.466
∆IL-23	0.671	< **0.001**	0.460	**0.005**	0.223	0.198	0.460	**0.005**
∆IFN-γ	0.271	0.086	0.253	0.111	0.256	0.106	-0.050	0.755
∆Resistin	0.034	0.831	0.064	0.691	-0.005	0.975	0.089	0.579
∆Leptin	0.554	< **0.001**	0.368	**0.019**	0.315	**0.048**	0.260	0.105
∆HMW adiponectin	-0.251	0.113	-0.113	0.481	-0.048	0.767	-0.396	**0.010**
∆Tot-adiponectin	-0.318	**0.043**	-0.189	0.237	-0.159	0.320	-0.158	0.324

∆ = difference between BL and M6

Spearman correlations between BL and M6

*BMI* body mass index, *CRP* c-reactive protein, *DAS28CRP* disease activity score, *DAPSA* disease activity in psoriatic arthritis, *HMW* high molecular weight, *IFN* interferon, *IL* interleukin, *TNF* tumor necrosis factor, *Tot* total

∆IL23 positively correlated with ∆DAS28CRP (r_S_ = 0.460, *p* = 0.005) (Table 3 and Supplementary Fig. 1B), ∆CRP and Δleptin (r_S_ = 0.818, *p* < 0.001) (Supplementary table 1, Supplementary Fig. 1A) and negatively correlated with Δtot-adiponectin (Supplementary table 1). ∆leptin was positively correlated with ∆DAPSA (Table 3), whereas ΔHMW adiponectin negatively correlated with ΔCRP. ΔIL-17 was positively correlated with ΔTNF-α, ΔIL-6 and ΔIL-12/IL-23 p40 and ∆IFN-γ (Supplementary table 1). Δleptin negatively correlated with Δtot-adiponectin (Supplementary table 1).

## Discussion

In this interventional study we explored the effects of weight loss treatment by VLED on the cytokine/adipokine profile in patients with PsA and obesity and matched controls. A 18.6% reduction of BL weight in patients with PsA was accompanied by significant decreases in serum IL-23 and leptin and a significant increase in serum tot-adiponectin and HMW adiponectin at M6, with similar results in controls. In the patients with PsA we also found significant correlations between a decrease in BMI with improvement of DAS28CRP, reductions in serum IL-23, IL-13, TNF-α, leptin and an increase in serum tot-adiponectin. Also, a correlation barely reaching significance (*p* = 0.042) between ∆BMI and ∆IL-17 was seen in patients with PsA. In controls, the serum levels of TNF-α, IL-1β, IL-6, IL-12/IL-23 p40, IL-23, IFN-γ and leptin, decreased significantly whereas serum tot-adiponectin and HMW adiponectin increased significantly at M6. This study builds upon our previously reported effects of weight loss treatment with VLED and concomitantly significantly reduced disease activity, as shown by reduced DAS28CRP and DAPSA scores at M6 [12] and highlights the importance of obesity in cytokine and adipokine production and the effects on PsA.

The white adipose tissue is an endocrine organ, consisting of adipocytes, immunological cells, that secrete hundreds of bioactive peptides, there among proinflammatory cytokines and adipokines, such as TNF-α, IL-17, IL-23, leptin, resistin and adiponectin that may be of importance in PsA [9–11]. In obesity, hypertrophy and subsequent necrosis of adipocytes occurs along with hypoxia and infiltration of macrophages with different phenotypes that interact with immune cells such as T-lymphocytes. A shift from an antiinflammatory (M2) adipose tissue macrophages (ATM) to pro-inflammatory (M1) ATM and promotion of IL-17 producing T-helper (Th)-17 cells through transforming growth factor (TGF)-β and IL-6 along with the stabilization of Th17 cells by IL-23 contribute to the proinflammatory state of obesity [21]. The white adipose tissue and localized fat pads inside joints, may play parts in developing enthesitis and PsA through cytokine and adipokine production [22]. IL-17 and IL-23, contribute to entheseal inflammation, arthritis and bone loss in PsA [23] and together with IL-22 among other cytokines, play a role in keratinocyte proliferation in PsO [24]. Inhibiting IL-17 and IL-23 has accordingly proven effective in PsO and PsA [24–26]. Increased serum levels of several proinflammatory cytokines compared to individuals without PsO was reported in a systematic review and meta-analysis [27]. In a review by Bianchi et al., comprising weight loss studies not assessing patients with PsA specifically, reductions in inflammatory markers such as leptin, CRP, IL-6 and TNF-α were reported after weight loss [28].

IL-23, a heterodimeric cytokine that consists of the subunits p19 (unique for IL-23) and p40 (shared subunit with IL-12) is produced by activated myeloid cells, mainly dendritic cells, monocytes and macrophages [23], but there are also cells within the enthesis, entheseal resident immune cells, capable of IL-17 and IL-23 production [29]. In our study, IL-23 was significantly reduced following weight loss in patients with PsA and controls. In patients with PsA, ∆IL-23 was strongly positively correlated with ∆BMI, ∆DAS28CRP and ∆leptin. In a recent study by Villarreal et al., not comprising patients with PsA specifically, lowered IL-23 levels but not significantly lowered IL-17 levels were reported six months after bariatric surgery in 32 patients with a preoperative median BMI of 41 [30]. Despite the importance of IL-23 in PsA, we found that serum IL-23 levels were surprisingly significantly lower in patients with PsA compared to controls at BL, even though only one of the patients with PsA reported treatment with the IL-12/IL-23 p40 inhibitor ustekinumab and none were treated with IL-17 inhibitors or IL-23 inhibitors. Possibly, the moderate disease activity in the patients with PsA and the higher BL BMI in controls can explain the higher BL IL-23 levels seen in controls. Speculatively, a reduction in serum IL-23 in PsA patients, although less pronounced compared to controls, may be more important in an IL-23 driven disease such as PsA compared to in those without PsA.

IL-17 is produced by several cells, including Th17 cells, γδ T cells, innate lymphoid cells and natural killer (NK) cells [23]. IL-17 production can occur dependently and independently of IL-23, but IL-23 has a promoting and stabilizing role on Th17 cells, increasing the pro-inflammatory potential of Th17 cells [23]. IL-17 producing cells have been found in greater numbers in synovial fluid in joints of patients with PsA compared with in patients with RA [31] as well as in PsO skin compared to normal skin [32]. The reductions in serum IL-17 in patients with PsA and controls at M6 were insignificant in the current study. However, a significant but modest correlation between ∆IL-17 and ∆BMI were seen in patients with PsA. As previously mentioned, many of the IL-17 samples were below the detection limit stated by the manufacturer, and therefore excluded, reducing the possibility of finding a significant difference between BL and M6 and thus leading to results that should be interpreted with caution and further explored in future studies in PsA.

We found a significant positive correlation between ∆BMI and ∆IL-13 in patients with PsA. IL-13, driven by a Th2 response rather than the Th1 and Th17 response seen in PsA, has generally not been considered to play a role in PsA. However, dupilumab, a monoclonal antibody that works by blocking the receptor of IL-4 and IL-13 (IL-4R1) and is used for treating asthma and atopic dermatitis, has been reported to be associated with side effects such as plaque PsO, generalized pustular PsO and nail PsO, speculatively an effect of increased production of IL-23 and consequently IL-17, due to blockade of the regulatory pathway of IL-4 [33].

TNF-α is secreted by activated macrophages, NK cells, lymphocytes, and adipose tissue [34] and increases production of IL-1 and IL-6 through the inflammation regulator nuclear factor kappa light-chain-enhancer of activated B cells (*NF*-*κB*) [35]. We found a significant positive correlation between ∆BMI and ∆TNF-α in patients with PsA. TNF-α is frequently elevated in obesity [34] and often reduced after weight loss in patients without rheumatic disease [28]. Serum levels of TNF-α decreased significantly in our control population, but not significantly in the patients with PsA. Whether the greater reductions in TNF-α and several other cytokines seen in controls as compared to patients with PsA could be explained by a greater weight loss in controls, absence of rheumatic disease, anti-rheumatic treatment, or the generally low serum cytokine levels in patients with PsA is a matter of speculation. In a study by Di Minno et al., comprising overweight/obese patients with PsA, started on treatment with TNF-α inhibitors along with a hypocaloric diet, a significant and dose-dependent effect of weight loss on the chance of achieving MDA at M6 was seen [6]. Although not measuring cytokines and adipokines in that study, it is plausible that weight loss in obese subjects with PsA can lead to a reduction in the systemic inflammatory burden and have a positive effect on disease activity partly mediated by a reduction in TNF-α.

Leptin, mainly produced in white adipose tissue and serum levels correlate with body fat [36] and leptin plays an important part in regulation of satiety and energy expenditure. Leptin has several effects on immune cells, including shifting T-lymphocytes towards a proinflammatory phenotype, increasing the number of Th17 cells, escalating proliferation of monocytes and augmenting the production of TNF-α, IL-1β, IL-6, IL-12 and IL-17 [37]. Many but not all of the weight loss studies identified in the review study by Bianchi et al. reported a reduction in leptin after weight loss [28]. We reported reduced serum leptin levels after weight loss in patients with PsA and controls, and ∆leptin positively correlated with ∆BMI and ∆DAS28CRP in the patients with PsA. In a meta-analysis by Kyriakou et al. higher serum leptin and resistin and lower serum adiponectin levels were reported in patients with PsO compared to controls [38]. In a study by Eder et al., serum leptin levels weakly correlated with active joint count in PsA (r = 0.1, *p* = 0.05) [39]. Higher leptin levels have been reported in women but not men (perhaps reflecting the larger proportion of fat mass in women as compared to men), and increased adiponectin levels have been seen in PsA compared to PsO [39].

Adiponectin is mainly produced by adipocytes and circulate in blood in three isoforms: low-molecular weight (LMW), medium-molecular weight (MMW) and HMW-adiponectin, the two latter being most abundant in the circulation. Pro- as well as anti-inflammatory roles depending on isoform ratio of adiponectin, effector site, binding receptor and disease studied have been proposed [40]. HMW-adiponectin has been suggested to be more pro-inflammatory than LMW-adiponectin [40]. In the current study, serum levels of tot-adiponectin and HMW-adiponectin were increased in patients with PsA and controls after weight loss and ∆tot-adiponectin negatively correlated with ∆BMI in the patients with PsA. Increased [41] as well as unchanged up to a 16% reduction of BL weight, but increased for greater weight loss than 16% increased as reported by Magkos et al. [42] levels of adiponectin have been reported after weight loss in studies not specifically addressing patients with PsA. Locally, in the joints of patients with RA, adiponectin has been suggested to have proinflammatory properties [43] and high adiponectin levels has been shown to be a predictor of future RA development in a long-term follow up study of individuals with obesity [44]. The multifaceted effects of adiponectin on inflammation and the inability to measure all forms of adiponectin in this study makes it difficult to draw conclusions regarding the role of adiponectin in PsA.

### Limitations and strengths

Some limitations should be addressed. First, this is not a randomized trial, and we did not include a control group with PsA that did not undergo a dietary intervention. We did, however, include a control group that underwent the same VLED treatment. The control group was without PsA, matched for sex, age and weight, although with higher BMI than the patients with PsA. Ideally, the patients with PsA should have been matched for BMI rather than weight, but due to a limited number of controls this was not possible (patients at the obesity center usually have a higher BMI compared to the patients with PsA in this study). Secondly, it cannot be ruled out that the effects on cytokines and adipokines are influenced by the temporary starvation. Nevertheless, at the sixth month visit most of the patients with PsA and controls had gained some weight compared with at the three months visit, suggesting a positive energy balance. Thirdly, the impact of physical activity on cytokines and adipokines was not assessed, although patients with PsA and controls received the same advice, which was in accordance with the general Swedish recommendations of physical activity. Fourthly, there were some methodological difficulties in measuring serum cytokine levels, some analytes being expressed at very low levels, just above detection limit, and concentrations determined from an extrapolated part, less reliable part of the standard curve. Patients with PsA and controls were, however, assessed in the same manner. Fifthly, serum levels of cytokines and adipokines may be an imprecise measure for the actual levels in entheses/joints/tissues where cytokines and adipokines exercise many of their effects. Sixthly, the small sample size (power estimations were originally not calculated for finding differences in cytokines and adipokines after weight loss, but for detecting a statistically significant difference in MDA at 10% weight loss with a significance level of *p* < 0.05 and a statistical power of 80%, which was calculated to ≥ 28 individuals in both treatment groups) could have precluded statistically significant findings. Also, the low cytokine levels, speculatively due to the moderate to low disease activity in patients with PsA might have diminished the differences in cytokine levels. On the other hand, including everyday care patients with PsA with moderate/low disease activity, representing a wide range of patients with PsA, increases the generalizability of the study. Patients treated with TNF-α inhibitors may have influenced the results regarding TNF-α. The large number of statistical tests performed increases the risk of false positive results and adjustments for multiple testing by for instance Bonferroni corrections could have been performed but we chose not to, due to the uncertainties/criticism that has been raised regarding if corrections for multiple testing should be performed or not [45]. Strengths of the study include the structured follow-up design, low attrition rate and successful weight loss intervention. Moreover, treatment with cs/bDMARDs were held unchanged from three month before BL until M6, minimizing the impact of treatment change on cytokines, adipokines and disease activity.

## Conclusions

Weight loss treatment with VLED in patients with PsA and obesity was accompanied by significant reductions in serum IL-23 and leptin and significant increases in serum tot-adiponectin and HMW adiponectin. The reduction in BMI was strongly positively correlated with the change in serum IL-23. The study supports important links between obesity both in general and in PsA through IL-23.

## Supplementary Information

**Additional file 1: Supplementary table 1.** Spearman correlations for ∆IL-23, ∆IL-17, ∆leptin and other analytes, between baseline and month 6 in patients with psoriatic arthritis, *n*=41. **Supplementary figure 1.** Scatterplots of the correlations between ΔIL-23 and a) Δleptin, b) ΔDAS28CRP, at month 6 in patients with psoriatic arthritis.

## Data Availability

The data analyzed in the current study are available from the corresponding author upon reasonable request.

## Abbreviations

ATM: Adipose tissue macrophage
BL: Baseline
BMI: Body mass index
BSA: Body surface area
B: Biologic
Caspar: Classification criteria for Psoriatic Arthritis
Cs: Conventional synthetic
DAPSA: Disease activity in psoriatic arthritis
DAS28CRP: Disease activity score
HAQ: Health assessment questionnaire
HMW: High molecular weight
IL: Interleukin
IQR: Interquartile range
LMW: Low molecular weight
MDA: Minimal disease activity
MMW: Medium molecular weight
M6: Month 6
PsA: Psoriatic arthritis
PsO: Psoriasis
TGF: Transforming growth factor
Th: T-helper
TNF: Tumor necrosis factor
Tot: Total
VAS: Visual analogue scale
VLED: Very low energy diet

## References

[1] Ritchlin CT, Colbert RA, Gladman DD (2017). Psoriatic Arthritis. N Engl J Med.

[2] Landgren AJ, Bilberg A, Eliasson B, Larsson I, Dehlin M, Jacobsson L, et al. Cardiovascular risk factors are highly overrepresented in Swedish patients with psoriatic arthritis compared with the general population. Scand J Rheumatol. 2019:1–5. 10.1080/03009742.2019.1672783 31631735

[3] Bhole VM, Choi HK, Burns LC, Vera Kellet C, Lacaille DV, Gladman DD (2012). Differences in body mass index among individuals with PsA, psoriasis. RA and the general population Rheumatology.

[4] Thomsen RS, Nilsen TI, Haugeberg G, Gulati AM, Kavanaugh A, Hoff M. Adiposity and physical activity as risk factors for developing psoriatic arthritis. Longitudinal data from the HUNT study. Arthritis Care Res (Hoboken). 2019. 10.1002/acr.24121 31811695

[5] Haroon M, Gallagher P, Heffernan E, FitzGerald O (2014). High prevalence of metabolic syndrome and of insulin resistance in psoriatic arthritis is associated with the severity of underlying disease. J Rheumatol.

[6] Di Minno MN, Peluso R, Iervolino S, Russolillo A, Lupoli R, Scarpa R (2014). Weight loss and achievement of minimal disease activity in patients with psoriatic arthritis starting treatment with tumour necrosis factor alpha blockers. Ann Rheum Dis.

[7] Eder L, Thavaneswaran A, Chandran V, Cook RJ, Gladman DD (2015). Obesity is associated with a lower probability of achieving sustained minimal disease activity state among patients with psoriatic arthritis. Ann Rheum Dis.

[8] Campanholo CB, Maharaj AB, Corp N, Bell S, Costa L, de Vlam K (2023). Management of Psoriatic Arthritis in Patients With Comorbidities: An Updated Literature Review Informing the 2021 GRAPPA Treatment Recommendations. J Rheumatol.

[9] Versini M, Jeandel PY, Rosenthal E, Shoenfeld Y (2014). Obesity in autoimmune diseases: not a passive bystander. Autoimmun Rev.

[10] Fasshauer M, Bluher M (2015). Adipokines in health and disease. Trends Pharmacol Sci.

[11] Ivanov S, Merlin J, Lee MKS, Murphy AJ, Guinamard RR (2018). Biology and function of adipose tissue macrophages, dendritic cells and B cells. Atherosclerosis.

[12] Klingberg E, Bilberg A, Bjorkman S, Hedberg M, Jacobsson L, Forsblad-d'Elia H (2019). Weight loss improves disease activity in patients with psoriatic arthritis and obesity: an interventional study. Arthritis Res Ther.

[13] Klingberg E, Björkman S, Eliasson B, Larsson I, Bilberg A (2020). Weight loss is associated with sustained improvement of disease activity and cardiovascular risk factors in patients with psoriatic arthritis and obesity: a prospective intervention study with two years of follow-up. Arthritis Res Ther.

[14] Taylor W, Gladman D, Helliwell P, Marchesoni A, Mease P, Mielants H (2006). Classification criteria for psoriatic arthritis: development of new criteria from a large international study. Arthritis Rheum.

[15] Healy PJ, Helliwell PS (2008). Measuring clinical enthesitis in psoriatic arthritis: assessment of existing measures and development of an instrument specific to psoriatic arthritis. Arthritis Rheum.

[16] Fries JF, Spitz P, Kraines RG, Holman HR (1980). Measurement of patient outcome in arthritis. Arthritis Rheum.

[17] Prevoo ML, van 't Hof MA, Kuper HH, van Leeuwen MA, van de Putte LB, van Riel PL. Modified disease activity scores that include twenty-eight-joint counts. Development and validation in a prospective longitudinal study of patients with rheumatoid arthritis. Arthritis Rheum. 1995;38(1):44–8. 10.1002/art.1780380107 7818570

[18] Schoels M, Aletaha D, Funovits J, Kavanaugh A, Baker D, Smolen JS (2010). Application of the DAREA/DAPSA score for assessment of disease activity in psoriatic arthritis. Ann Rheum Dis.

[19] Bożek A, Reich A (2017). The reliability of three psoriasis assessment tools: Psoriasis area and severity index, body surface area and physician global assessment. Advances in clinical and experimental medicine : official organ Wroclaw Medical University.

[20] Coates LC, Fransen J, Helliwell PS (2010). Defining minimal disease activity in psoriatic arthritis: a proposed objective target for treatment. Ann Rheum Dis.

[21] Chehimi M, Vidal H, Eljaafari A. Pathogenic Role of IL-17-Producing Immune Cells in Obesity, and Related Inflammatory Diseases. Journal of clinical medicine. 2017;6(7). 10.3390/jcm6070068 PMC5532576 28708082

[22] Kruglikov IL, Wollina U (2017). Local effects of adipose tissue in psoriasis and psoriatic arthritis. Psoriasis (Auckland, NZ).

[23] Gravallese EM, Schett G (2018). Effects of the IL-23-IL-17 pathway on bone in spondyloarthritis. Nat Rev Rheumatol.

[24] Ghoreschi K, Balato A, Enerbäck C, Sabat R (2021). Therapeutics targeting the IL-23 and IL-17 pathway in psoriasis. Lancet.

[25] Sakkas LI, Zafiriou E, Bogdanos DP. Mini Review: New Treatments in Psoriatic Arthritis. Focus on the IL-23/17 Axis. Front Pharmacol. 2019;10:872. 10.3389/fphar.2019.00872 PMC6691125 31447673

[26] Yang K, Oak ASW, Elewski BE (2021). Use of IL-23 Inhibitors for the Treatment of Plaque Psoriasis and Psoriatic Arthritis: A Comprehensive Review. Am J Clin Dermatol.

[27] Bai F, Zheng W, Dong Y, Wang J, Garstka MA, Li R (2018). Serum levels of adipokines and cytokines in psoriasis patients: a systematic review and meta-analysis. Oncotarget.

[28] Bianchi VE (2018). Weight loss is a critical factor to reduce inflammation. Clinical nutrition ESPEN.

[29] Bridgewood C, Sharif K, Sherlock J, Watad A, McGonagle D (2020). Interleukin-23 pathway at the enthesis: The emerging story of enthesitis in spondyloarthropathy. Immunol Rev.

[30] Villarreal-Calderon JR, Cuellar-Tamez R, Castillo EC, Luna-Ceron E, García-Rivas G, Elizondo-Montemayor L (2021). Metabolic shift precedes the resolution of inflammation in a cohort of patients undergoing bariatric and metabolic surgery. Sci Rep.

[31] Menon B, Gullick NJ, Walter GJ, Rajasekhar M, Garrood T, Evans HG (2014). Interleukin-17+CD8+ T cells are enriched in the joints of patients with psoriatic arthritis and correlate with disease activity and joint damage progression. Arthritis & rheumatology (Hoboken, NJ).

[32] Res PC, Piskin G, de Boer OJ, van der Loos CM, Teeling P, Bos JD (2010). Overrepresentation of IL-17A and IL-22 producing CD8 T cells in lesional skin suggests their involvement in the pathogenesis of psoriasis. PLoS ONE.

[33] Jaulent L, Staumont-Sallé D, Tauber M, Paul C, Aubert H, Marchetti A (2021). De novo psoriasis in atopic dermatitis patients treated with dupilumab: a retrospective cohort. J Eur Acad Dermatol Venereol.

[34] Phillips CL, Grayson BE (2020). The immune remodel: Weight loss-mediated inflammatory changes to obesity. Exp Biol Med (Maywood).

[35] Lee J (2013). Adipose tissue macrophages in the development of obesity-induced inflammation, insulin resistance and type 2 diabetes. Arch Pharm Res.

[36] Considine RV, Sinha MK, Heiman ML, Kriauciunas A, Stephens TW, Nyce MR (1996). Serum immunoreactive-leptin concentrations in normal-weight and obese humans. N Engl J Med.

[37] Hwang J, Yoo JA, Yoon H, Han T, Yoon J, An S (2021). The Role of Leptin in the Association between Obesity and Psoriasis. Biomol Ther (Seoul).

[38] Kyriakou A, Patsatsi A, Sotiriadis D, Goulis DG (2018). Serum Leptin, Resistin, and Adiponectin Concentrations in Psoriasis: A Meta-Analysis of Observational Studies. Dermatology (Basel).

[39] Eder L, Jayakar J, Pollock R, Pellett F, Thavaneswaran A, Chandran V (2013). Serum adipokines in patients with psoriatic arthritis and psoriasis alone and their correlation with disease activity. Ann Rheum Dis.

[40] Choi HM, Doss HM, Kim KS. Multifaceted Physiological Roles of Adiponectin in Inflammation and Diseases. Int J Mol Sci. 2020;21(4). 10.3390/ijms21041219 PMC7072842 32059381

[41] Christiansen T, Paulsen SK, Bruun JM, Ploug T, Pedersen SB, Richelsen B (2010). Diet-induced weight loss and exercise alone and in combination enhance the expression of adiponectin receptors in adipose tissue and skeletal muscle, but only diet-induced weight loss enhanced circulating adiponectin. J Clin Endocrinol Metab.

[42] Magkos F, Fraterrigo G, Yoshino J, Luecking C, Kirbach K, Kelly SC (2016). Effects of Moderate and Subsequent Progressive Weight Loss on Metabolic Function and Adipose Tissue Biology in Humans with Obesity. Cell Metab.

[43] Giles JT, van der Heijde DM, Bathon JM (2011). Association of circulating adiponectin levels with progression of radiographic joint destruction in rheumatoid arthritis. Ann Rheum Dis.

[44] Zhang Y, Peltonen M, Andersson-Assarsson JC, Svensson PA, Herder C, Rudin A, et al. Elevated adiponectin predicts the development of rheumatoid arthritis in subjects with obesity. Scand J Rheumatol. 2020:1–9. 10.1080/03009742.2020.1753808 PMC7666010 32667228

[45] Perneger TV (1998). What's wrong with Bonferroni adjustments. BMJ.

