# Supplementary material for: Serum IL-23 significantly decreased in obese patients with psoriatic arthritis six months after a structured weight loss intervention
Source: Arthritis Res Ther. 2023 Jul 27;25:131. doi: 10.1186/s13075-023-03105-8 (PMC10373368; doi:10.1186/s13075-023-03105-8)
Supplement: Supplementary file 1 — Additional file 1: Supplementary table 1. Spearman correlations for ∆IL-23, ∆IL-17, ∆leptin and other analytes, between baseline and month 6 in patients with psoriatic arthritis, n=41. Supplementary figure 1. Scatterplots of the correlations between ΔIL-23 and a) Δleptin, b) ΔDAS28CRP, at month 6 in patients with psoriatic arthritis. [file 13075_2023_3105_MOESM1_ESM.docx]

|  |
| --- |

Supplementary table 1, Spearman correlations for ∆IL-23, ∆IL-17, ∆leptin and other analytes, between baseline and month 6 in patients with psoriatic arthritis, n=41

|  | ∆IL-23 | p-value | ∆IL-17 | p-value | ∆leptin | p-value |
| --- | --- | --- | --- | --- | --- | --- |
| ∆CRP | 0.460 | **0.005** | 0.204 | 0.466 | 0.260 | 0.105 |
| ∆TNF-α | 0.242 | 0.161 | 0.520 | **0.047** | 0.208 | 0.198 |
| ∆IL-1β | 0.107 | 0.636 | 0.419 | 0.200 | 0.210 | 0.302 |
| ∆IL-6 | 0.136 | 0.437 | 0.584 | **0.022** | 0.037 | 0.820 |
| ∆IL-8 | -0.276 | 0.109 | 0.120 | 0.670 | -0.235 | 0.144 |
| ∆IL-12/IL-23 p40 | 0.188 | 0.320 | 0.695 | **0.006** | 0.241 | 0.170 |
| ∆IL-13 | 0.164 | 0.353 | 0.501 | 0.057 | 0.210 | 0.200 |
| ∆IL-17 | 0.420 | 0.135 |  |  | 0.282 | 0.308 |
| ∆IL-23 |  |  | 0.420 | 0.135 | 0.818 | **<0.001** |
| ∆IFN-γ | 0.109 | 0.534 | 0.600 | **0.018** | 0.146 | 0.368 |
| ∆Resistin | -0.146 | 0.404 | 0.093 | 0.742 | -0.200 | 0.216 |
| ∆Leptin | 0.818 | **<0.001** | 0.282 | 0.308 |  |  |
| ∆HMW adiponectin | -0.321 | 0.060 | 0.080 | 0.777 | -0.223 | 0.166 |
| ∆Tot-adiponectin | -0.355 | **0.036** | -0.211 | 0.450 | -0.340 | **0.032** |

| BMI body mass index  CRP c-reactive protein  HMW high molecular weight  IFN interferon  IL interleukin |
| --- |
| TNF tumor necrosis factor  Tot total  VAS visual analogue scale |

Supplementary figure 1, Scatterplots of the correlations between ∆IL-23 and a) ∆leptin, b) ∆DAS28CRP, at month 6 in patients with psoriatic arthritis (PsA)

| 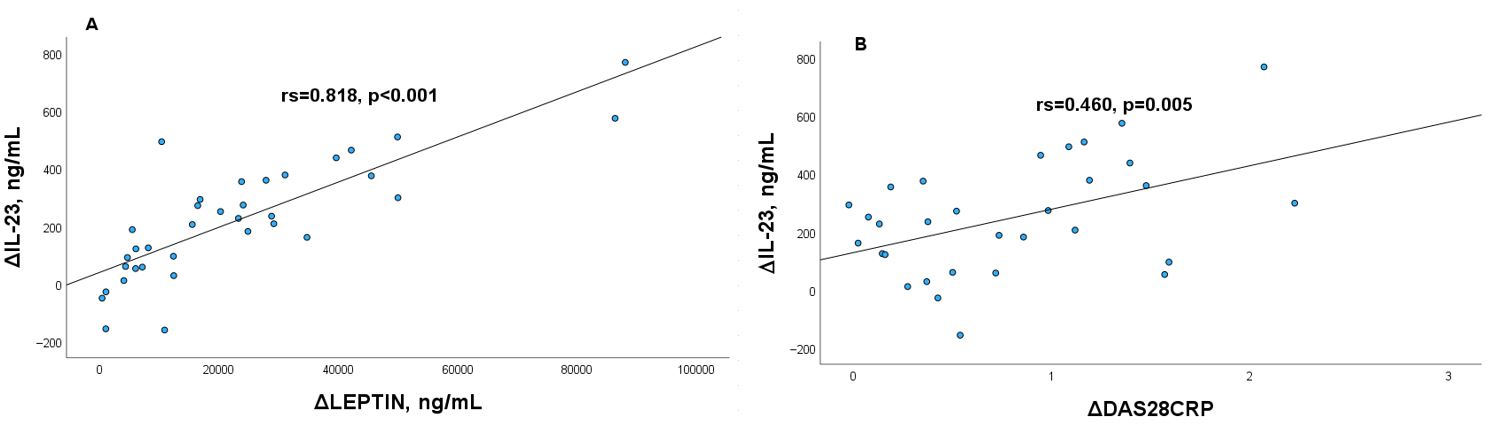 |  |  |  |  |  |  |  |  |  |  |  |  |  |  |  |
| --- | --- | --- | --- | --- | --- | --- | --- | --- | --- | --- | --- | --- | --- | --- | --- |

DAS28CRP disease activity score

IL interleukin
